# Analysis and reporting of trials which randomise couples to therapy based interventions: how should we do it?

**DOI:** 10.1186/1745-6215-16-S2-P6

**Published:** 2015-11-16

**Authors:** Stephen Walters, Ellen Lee

**Affiliations:** 1 University of Sheffield, Sheffield, UK

## 

The evaluation of therapy-based interventions often involves interventions which are delivered to both couples and individuals (e.g. parenting classes).

In these circumstances; it seems ly that the effectiveness of the intervention could depend on the skill of the therapist delivering it; as well as the interactions between the couples receiving the therapy. This leads to a potential clustering of the outcomes for the participants being treated by the same therapist and within the same couple.

If the outcomes are clustered then the usual statistical methods for analysing RCT data to compare outcomes between the intervention and control groups, may not be appropriate as they assume that the observed outcomes on different participants are independent.

The Lifestyle Matters (LM) trial is a pragmatic, two-arm, parallel group, individually randomised controlled trial, to evaluate the effectiveness of the LM intervention, to promote healthy ageing, compared to usual care in people aged 65 or more.

The Lifestyle Matters intervention consists of up to 16 weekly group (size 8 to 16 participants) sessions and four individual sessions with a facilitator.

The Lifestyle matters study randomised 288 individuals (145 Intervention: 143 control) of whom 18 were couples (9 Intervention: 9 Control) and followed them up for 24 months.

Using the Lifestyle Matters trial as an example this talk will describe and compare the different ways of analysing (including multilevel mixed-effects regression models) and reporting trials which randomise individuals and couples to therapy based interventions.

